# PAM50 subtyping and ROR score add long-term prognostic information in premenopausal breast cancer patients

**DOI:** 10.1038/s41523-022-00423-z

**Published:** 2022-05-09

**Authors:** Christine Lundgren, Pär-Ola Bendahl, Sarah E. Church, Maria Ekholm, Mårten Fernö, Carina Forsare, Ute Krüger, Bo Nordenskjöld, Olle Stål, Lisa Rydén

**Affiliations:** 1 Department of Oncology, Region Jönköping County Jönköping, Sweden; 2 Department of Clinical Sciences Lund, Division of Oncology, Lund University, Lund, Sweden; 3 NanoString Technologies, Inc., Seattle, United States; 4 Sahlgrenska Center for Cancer Research, Department of Laboratory Medicine, Institute of Biomedicine, Sahlgrenska Academy at University of Gothenburg, Gothenburg, Sweden; 5 Department of Biomedical and Clinical Sciences, Linköping University, Linköping, Sweden; 6 Department of Clinical Sciences Lund, Division of Surgery, Lund University, Lund, Sweden; 7 Department of Surgery, Skåne University Hospital, Malmö, Sweden

**Keywords:** Breast cancer, Tumour biomarkers, Predictive markers, Prognostic markers

## Abstract

PAM50 intrinsic subtyping and risk of recurrence (ROR) score are approved for risk profiling in postmenopausal women. We aimed to examine their long-term prognostic value in terms of breast cancer-free interval (BCFi) and overall survival (OS) (*n* = 437) in premenopausal women randomised to 2 years of tamoxifen versus no systemic treatment irrespective of hormone-receptor status. Intrinsic subtyping added independent prognostic information in patients with oestrogen receptor-positive/human epidermal growth factor 2-negative tumours for BCFi and OS after maximum follow-up (overall *P*-value 0.02 and 0.006, respectively) and those with high versus low ROR had worse prognosis (maximum follow-up: hazard ratio (HR)_BCFi_: 1.70, *P* = 0.04). The prognostic information by ROR was similar regarding OS and in multivariable analysis. These results support that PAM50 subtyping and ROR score provide long-term prognostic information in premenopausal women. Moreover, tamoxifen reduced the incidence of breast cancer events only in patients with Luminal A_PAM50_ tumours (0–10 years: HR_BCFi(Luminal A)_: 0.41, HR_BCFi(Luminal B)_: 1.19, *P*_interaction_ = 0.02).

**Trial registration:** This trial is registered in the ISRCTN database, trial ID: ISRCTN12474687.

## Introduction

The classification of breast cancer tumours by gene expression analysis into intrinsic subtypes (Luminal A, Luminal B, human epidermal growth factor receptor 2-enriched [HER2-E], and Basal-like), is well-established^1–3^. Genomic testing is recommended as a complement to conventional risk assessment in postmenopausal patients with equivocal risk of recurrence^4,5^. PAM50 intrinsic subtyping and risk of recurrence (ROR) score, initially developed by Parker et al., are included in the Prosigna^©^ Breast Cancer Prognostic Gene Signature Assay^3,6^. This is approved and validated for postmenopausal women with oestrogen receptor-positive/HER2-negative (ER+/HER2−) tumours allocated to 5 years of endocrine therapy, and ROR score provides prognostic information^7–11^.

The prognostic value of PAM50 subtypes and ROR score in premenopausal women remains unclear. Previous studies have indicated that PAM50 and ROR score are applicable also for premenopausal women^12–15^. Moreover, some of these studies also demonstrated a possible predictive effect of PAM50 subtypes for tamoxifen and chemotherapy benefit^12,13^. Surrogate classification of breast cancer tumours into Luminal A_Surrogate Classification, (SC)_ and Luminal B_SC_, using immunohistochemistry (IHC)/in situ hybridisation (ISH), was used in clinical settings before multigene assays were broadly implemented for prognostication and guiding decisions about adjuvant treatments. However, surrogate classifications have shown poor concordance to the corresponding intrinsic subtypes^16–19^, resulting in suboptimal risk estimation for patients with ER+/HER2− tumours.

In the SBII:2pre trial, premenopausal women were randomised between 2 years of adjuvant tamoxifen or no adjuvant systemic therapy (control), irrespective of hormone-receptor status and thus one third of the patients had ER-negative tumours. Availability of long-term follow-up data (>30 years) and preserved archival tumour tissues from the participants allowed us to assess the long-term effects across gene expression profiles and subtypes in this cohort.

The primary aim of this study was to investigate the prognostic value of PAM50 intrinsic subtypes and ROR score in premenopausal patients with ER+/HER2− tumours. The secondary aims were to compare luminal PAM50 and St. Gallen 2013 surrogate subtypes and to evaluate if luminal PAM50 subtypes can be used to predict tamoxifen benefit in premenopausal patients.

## Results

### Sample availability and cohort characteristics

**Fig. 1:**
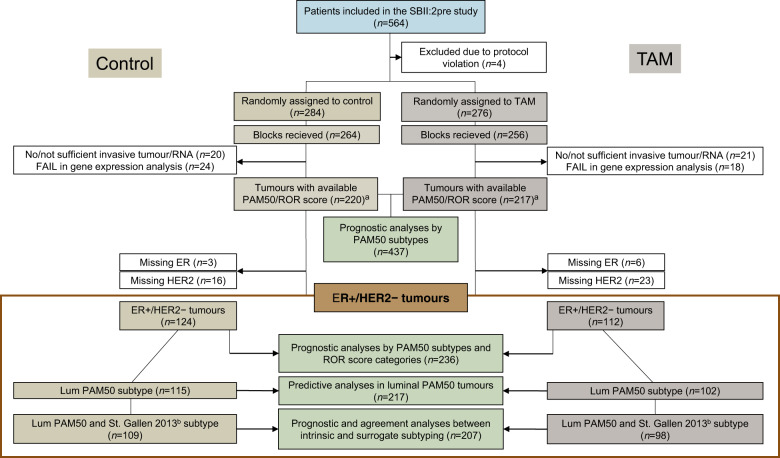
Flow chart of the included patients. ^a^Available ROR score categories in *n* = 219 and *n* = 216 patients in the control (no systemic treatment) and tamoxifen arm, respectively, due to *n* = 1 missing nodal status for one patient in each treatment arm. ^b^Defined accordingly: LumA_SC_, low Ki67 (<20%) and high PR (≥20%); LumB_SC_, high Ki67 (≥20%) and/or low PR (<20%). Cases with missing re-evaluated PR data were substituted (*n* = 2 in the control arm) with previously available IHC data for PR. ER oestrogen receptor, HER2 human epidermal growth factor receptor 2, IHC immunohistochemistry, Lum Luminal, PR progesterone receptor, ROR risk of recurrence, SC surrogate classification, TAM tamoxifen.

Gene expression analyses were successfully performed for 220 and 217 tumours (and the corresponding number of patients) in the control and tamoxifen arms, respectively (Fig. 1). The median follow-up time for those with no breast cancer events regarding breast cancer-free interval (BCFi) and overall survival (OS) were 28 and 33 years, respectively. Patient and tumour characteristics for the entire cohort and for the ER+/HER2− cohort are presented in Table 1.

**Table 1:** Patient and tumour characteristics for the whole study cohort (*n* = 560) by study arm and for the ER-positive/HER2-negative subgroup.

	Initial study cohort (*n* = 560)		ER+/HER2− cohort (*n* = 280)	
Characteristics	Control group *n* (%)	TAM-treated group *n* (%)	Control group *n* (%)	TAM-treated group *n* (%)
*Follow-up BCFi/OS (years)*^*a*^				
Median	28/33	28/33	28/33	28/33
Range (10th–90th percentiles)	(25–31)/(30–35)	(25–30)/(31–35)	(26–31)/(30–35)	(25–30)/(30–35)
*Age (years)*				
Median	45	45	46	46
Range	27–58	26–57	27–54	33–57
<40	59 (21)	51 (19)	24 (16)	17 (13)
≥40	225 (79)	225 (82)	124 (84)	115 (87)
*Tumour size (mm)*				
Median	22	25	22	23
Range	2–50	5–75	2–50	8–50
≤20	121 (43)	86 (31)	70 (47)	48 (37)
>20	163 (57)	189 (69)	78 (53)	83 (63)
Missing	0	1	0	1
*Nodal status*				
Median number of positive nodes	1	1	1.5	1
Range	0–22	0–21	0–15	0–17
Node-negative	75 (27)	83 (30)	35 (24)	36 (27)
Node-positive	208 (74)	192 (70)	113 (76)	96 (73)
Missing	1	1	0	0
*NHG*				
1	32 (12)	27 (11)	25 (17)	22 (17)
2	115 (44)	105 (42)	88 (60)	68 (53)
3	116 (44)	117 (47)	33 (23)	39 (30)
Missing	21	27	2	3
*ER*				
Positive	191 (70)	171 (65)	148 (100)	132 (100)
Negative	84 (31)	91 (35)	0	0
Missing	9	14	0	0
*PR*				
Positive	185 (67)	163 (61)	139 (94)	118 (90)
Negative	92 (33)	103 (39)	9 (6)	13 (10)
Missing	7	10	0	1
*HER2*				
Negative	203 (84)	197 (87)	148 (100)	132 (100)
Positive	38 (16)	30 (13)	0	0
Missing	43	49	0	0
*LVI*				
Absent	138 (56)	124 (52)	75 (55)	64 (53)
Present	109 (44)	113 (48)	62 (45)	56 (47)
Missing	37	39	11	12
*Ki67 (%*)				
<14	18 (8)	25 (11)	13 (10)	18 (16)
14–19	25 (11)	27 (12)	22 (18)	15 (13)
≥20	184 (81)	167 (76)	91 (72)	82 (71)
Missing	57	57	22	17
*TILs (%)*				
<10	129 (52)	123 (52)	90 (66)	86 (72)
10–49	86 (35)	75 (32)	36 (26)	29 (24)
50–74	27 (11)	31 (13)	11 (8)	5 (4)
≥75	7 (3)	8 (3)	0	0
Missing	35	39	11	12
*Histopathological type*				
Ductal/NST	209 (84)	200 (83)	123 (84)	111 (84)
Lobular	22 (9)	21 (9)	18 (12)	14 (11)
Medullary	14 (6)	11 (5)	2 (1)	1 (1)
Other	5 (2)	10 (4)	3 (2)	6 (5)
Missing	34	34	2	0
*Subtype (IHC/ISH)*				
Luminal/HER2	148 (64)	132 (61)	148 (100)	132 (100)
HER2+	38 (16)	30 (14)	0	0
TNBC	46 (20)	54 (25)	0	0
Missing	52	60	0	0
*PAM50 intrinsic subtype*				
LumA	101 (46)	90 (42)	82 (66)	66 (59)
LumB	41 (19)	42 (19)	33 (27)	36 (32)
HER2-E	39 (18)	35 (16)	8 (7)	4 (4)
Basal-like	39 (18)	50 (23)	1 (1)	6 (5)
Missing	64	59	24	20
*ROR score*^*b*^				
Median	56	56	45	50
Range	0–94	1–94	4–94	12–94
Low	22 (10)	23 (11)	16 (13)	15 (13)
Intermediate	48 (22)	55 (26)	35 (28)	29 (26)
High	149 (68)	138 (64)	73 (59)	68 (61)
Missing	65	60	24	20
*N0 (node-negative)*^*c*^				
Low (0–40)	19 (33)	20 (32)	15 (52)	14 (45)
Intermediate (41–60)	11 (19)	21 (34)	6 (21)	7 (23)
High (61–100)	27 (47)	21 (34)	8 (28)	10 (32)
Missing	18	21	6	5
*N1 (1–3 positive nodes)*^*c*^				
Low (0–15)	3 (3)	3 (3)	1 (2)	1 (2)
Intermediate (16–40)	37 (35)	34 (32)	29 (45)	22 (38)
High (41–100)	65 (62)	71 (66)	35 (54)	35 (60)
Missing	34	28	12	11
*N2 (≥4 positive nodes)*^*c*^				
High (0–100)	57 (100)	46 (100)	30 (100)	23 (100)
Missing	12	10	6	4

*BCFi* breast cancer-free interval, *ER* oestrogen receptor, *HER2-E* human epidermal growth factor receptor 2-enriched, *IHC* immunohistochemistry, *ISH* in situ hybridisation, *Lum* Luminal, *LVI* lymphovascular invasion, *NHG* Nottingham histological grade, *NST* no special type, *OS* overall survival, *PR* progesterone receptor, *ROR* risk of recurrence, *TAM* tamoxifen, *TILs* tumour infiltrating lymphocytes, *TNBC* triple-negative breast cancer.

^a^Patients without events.

^b^The ROR score categories were defined by the following cut-offs based on N-status; N0; low: 0–40, intermediate: 41–60, high: 61–100, N1; low: 0–15, intermediate: 15–40, high: 41–100, N2; high: 0–100.

^c^ROR score stratified by nodal status.

The proportions of Luminal A, Luminal B, HER2-E, and Basal-like intrinsic subtypes by PAM50 (*n* = 437) were 44%, 19%, 17%, and 20%, respectively (Supplementary Fig. 1). The median ROR score was 56 and the proportions among patients with available nodal status classified into the low, intermediate, and high ROR categories (*n* = 435) were 10%, 24%, and 66%, respectively.

### Prognostic value of PAM50 subtypes

**Fig. 2:**
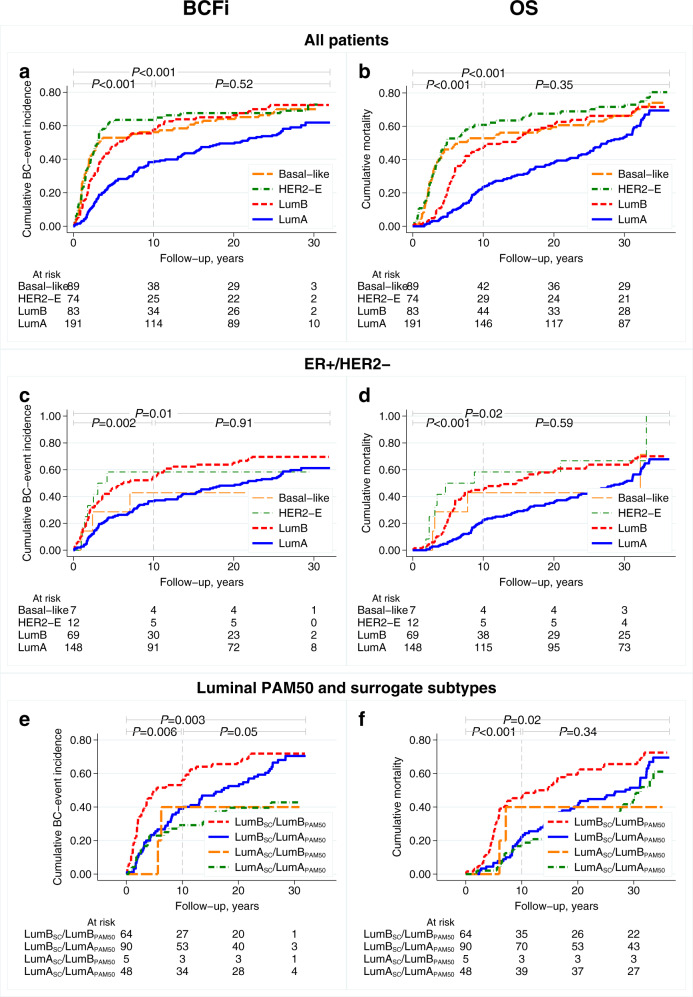
Cumulative incidence curves for BCFi and OS by PAM50 subtypes. (**a**, **b**) All included patients, (**c**, **d**) patients with ER-positive/HER2-negative tumours, and (**e**, **f**) patients with available intrinsic PAM50 and surrogate subtyping by St. Gallen 2013. Overall *P*-values from log rank test, Gehan’s version for BCFi, for maximum follow-up and for different time intervals. BCFi breast cancer-free interval, HER2-E human epidermal growth factor receptor 2-enriched, Lum Luminal, OS overall survival, SC surrogate classification.

Cumulative incidence curves for BCFi and OS by PAM50 subtypes are presented in Fig. 2a, b for all patients and in Fig. 2c, d for patients with ER+/HER2− tumours. After the maximum follow-up period, patients with ER+/HER2− and Luminal B_PAM50_ tumours had a higher cumulative incidence of breast cancer events than patients whose tumours were categorised as Luminal A_PAM50_ (hazard ratio [HR]_BCFi_: 1.56, 95% confidence interval [CI] 1.09–2.22, *P* = 0.01). The results were similar for the period 0–10 years (HR_BCFi_: 1.93, 95% CI: 1.27–2.93, *P* = 0.002; Table 2). The results also indicated increased overall mortality for patients whose tumours were Luminal B_PAM50_ as compared to Luminal A_PAM50_ (maximum follow-up: HR_OS_: 1.49, 95% CI: 1.05–2.12, *P* = 0.03; 0–10 years: HR_OS_: 2.55, 95% CI: 1.56–4.17, *P* < 0.001). The results were similar after adjusting for other clinicopathological variables (Table 2) and for all included patients irrespective of hormone-receptor status (Supplementary Table 1). Additionally, cumulative incidence curves for recurrence-free interval (RFi) with essentially the same results are depicted in Supplementary Fig. 2a–d.

**Table 2:** Cox regression uni- and multivariable models for BCFi and OS by PAM50 subtypes and St. Gallen 2013 surrogate subtypes for different time intervals in patients with ER-positive/HER2-negative tumours.

	Univariable		Multivariable^a^	
	BCFi	OS	BCFi	OS
	HR (95% CI); *P*-value			
*PAM50 subtype* *(ER+/HER2− cohort)*	0–10 years			
	(*n* = 236, *n* = 102 events) overall *P*-value = 0.01^d^	(*n* = 236, *n* = 74 events) overall *P*-value < 0.001^d^	(*n* = 233, *n* = 102 events) overall *P*-value = 0.04^d^	(*n* = 233, *n* = 74 events) overall *P*-value = 0.01^d^
LumA (Ref.)	1.00	1.00	1.00	1.00
LumB	1.93 (1.27–2.93); 0.002	2.55 (1.56–4.17); <0.001	2.02 (1.26–3.26); 0.004	2.42 (1.37–4.28); 0.002
HER2-E	2.22 (1.01–4.91); 0.05	4.34 (1.92–9.85); <0.001	1.74 (0.69–4.40); 0.24	3.35 (1.25–8.98); 0.02
Basal-like	1.25 (0.39–4.04); 0.70	2.38 (0.72–7.85); 0.16	1.17 (0.34–4.03); 0.80	1.67 (0.46–6.00); 0.43
	>10 years^b^			
	(*n* = 130, *n* = 43 events)	(*n* = 162, *n* = 77 events) overall *P*-value = 0.74^d^	(*n* = 127, *n* = 42 events)	(*n* = 159, *n* = 77 events) overall *P*-value = 0.31^d^
LumA (Ref.)	1.00	1.00	1.00	1.00
LumB	0.92 (0.45–1.87); 0.81	0.86 (0.50–1.48); 0.58	1.91 (0.79–4.59); 0.15	1.66 (0.87–3.16); 0.12
HER2-E	–	0.76 (0.18–3.12); 0.70	–	3.50 (0.63–19.45); 0.15
Basal-like	–	0.38 (0.05–2.76); 0.34	–	2.36 (0.25–22.11); 0.45
	Maximum follow-up time^c^			
	(*n* = 236, *n* = 145 events) overall *P*-value = 0.08^d^	(*n* = 236, *n* = 151 events) overall *P*-value = 0.05^d^	(*n* = 233, *n* = 144 events) overall *P*-value = 0.02^d^	(*n* = 233, *n* = 151 events) overall P-value = 0.006^d^
LumA (Ref.)	1.00	1.00	1.00	1.00
LumB	1.56 (1.09–2.22); 0.01	1.49 (1.05–2.12); 0.03	1.89 (1.25–2.86); 0.003	1.93 (1.28–2.92); 0.002
HER2-E	1.35 (0.62–2.93); 0.45	2.11 (1.06–4.20); 0.04	1.50 (0.62–3.67); 0.37	3.04 (1.33–6.95); 0.008
Basal-like	0.73 (0.23–2.30); 0.59	1.05 (0.38–2.87); 0.93	0.82 (0.24–2.74); 0.75	1.45 (0.49–4.28); 0.50
*St. Gallen 2013/PAM50 subtype*	0–10 years			
	(*n* = 207, *n* = 87 events) overall *P*-value = 0.007^d^	(*n* = 207, *n* = 60 events) overall *P*-value = 0.001^d^	(*n* = 205, *n* = 87 events) overall *P*-value = 0.04^d^	(*n* = 205, *n* = 60 events) overall *P*-value = 0.03^d^
LumB_SC_/LumB_PAM50_ (Ref.)	1.00	1.00	1.00	1.00
LumB_SC_/LumA_PAM50_	0.52 (0.33–0.83); 0.006	0.37 (0.21–0.66); 0.001	0.50 (0.29–0.84); 0.009	0.38 (0.20–0.74); 0.004
LumA_SC_/LumB_PAM50_	0.49 (0.12–2.05); 0.33	0.76 (0.18–3.20); 0.71	0.77 (0.18–3.32); 0.72	1.12 (0.25–5.07); 0.88
LumA_SC_/LumA_PAM50_	0.39 (0.21–0.73); 0.003	0.32 (0.15–0.68); 0.003	0.45 (0.23–0.91); 0.03	0.44 (0.19–1.01); 0.05
	>10 years^b^			
	(*n* = 117, *n* = 42 events) overall *P*-value = 0.06^e^	(*n* = 147, *n* = 70 events) overall *P*-value = 0.56^e^	(*n* = 115, *n* = 41 events) overall *P*-value = 0.06^e^	(*n* = 145, *n* = 70 events) overall *P*-value = 0.56^e^
LumB_SC_/LumB_PAM50_ (Ref.)	1.00	1.00	1.00	1.00
LumB_SC_/LumA_PAM50_	1.30 (0.62–2.69); 0.49	1.11 (0.62–1.98); 0.72	0.64 (0.26–1.60); 0.34	0.59 (0.30–1.15); 0.12
LumA_SC_/LumB_PAM50_	–	–	–	–
LumA_SC_/LumA_PAM50_	0.45 (0.16–1.23); 0.12	0.81 (0.41–1.59); 0.54	0.26 (0.08–0.80); 0.02	0.38 (0.18–0.84);0.02
	Maximum follow-up time^c^			
	(*n* = 207, *n* = 129 events) overall *P*-value = 0.004^d^	(*n* = 207, *n* = 130 events) overall *P*-value = 0.02^d^	(*n* = 205, *n* = 128 events) overall *P*-value = 0.01^d^	(*n* = 205, *n* = 130 events) overall *P*-value = 0.008^d^
LumB_SC_/LumB_PAM50_ (Ref.)	1.00	1.00	1.00	1.00
LumB_SC_/LumA_PAM50_	0.70 (0.47–1.02); 0.06	0.65 (0.44–0.96); 0.03	0.58 (0.37–0.90); 0.02	0.50 (0.32–0.79); 0.003
LumA_SC_/LumB_PAM50_	0.34 (0.08–1.41); 0.14	0.37 (0.09–1.52); 0.17	0.52 (0.12–2.24); 0.38	0.48 (0.11–2.05): 0.32
LumA_SC_/LumA_PAM50_	0.39 (0.23–0.67); 0.001	0.51 (0.32–0.83); 0.007	0.39 (0.21–0.70); 0.002	0.43 (0.25–0.74); 0.003

*BCFi* breast cancer-free interval, *CI* confidence interval, *ER* oestrogen receptor, *HER2-E* human epidermal growth factor receptor 2-enriched, *HR* hazard ratio, *Lum* Luminal, *NHG* Nottingham histological grade, *OS* overall survival, *SC* surrogate classification.

^a^All analyses are stratified by study region and adjusted for age (continuous), tumour size (>20 vs ≤20 mm), NHG (1 vs 2 vs 3), nodal status (N0 vs N1 vs N2) and treatment arm.

^b^From year 10 to maximum follow-up time.

^c^32 and 36 years regarding BCFi and OS, respectively.

^d^Overall *P*-value, three degree of freedom Wald test.

^e^Overall *P*-value, two degree of freedom Wald test.

### Agreement and prognostic effect of luminal PAM50 and St. Gallen 2013 surrogate subtypes

In the agreement analyses (ER + /HER2 − cohort, *n* = 207), 67% and 33% were assessed as Luminal A_PAM50_ and Luminal B_PAM50_, respectively. The corresponding figures for St. Gallen 2013 surrogate subtypes were 26% and 74%, respectively. In total, 58% (90/154) of patients classified as Luminal B_SC_ were classified as Luminal A_PAM50_ (Table 3).

**Table 3:** Distribution of luminal subtypes according to PAM50 and St. Gallen 2013 surrogate subtyping (*n* = 207) and corresponding agreement analyses (percentage and kappa [κ] statistic).

PAM50 subtype	St. Gallen 2013 surrogate subtyping *n* (%)	
	LumA_SC_ (*n* = 53)	LumB_SC_ (*n* = 154)
LumA_PAM50_ (*n* = 138)	48 (91)	90 (58)
LumB_PAM50_ (*n* = 69)	5 (9)	64 (42)
*Agreement (%)*	54	
*Kappa (κ) (95% CI)*	0.21 (0.12–0.30)	

*CI* confidence interval, *Lum* Luminal, *SC* surrogate classification.

The difference in outcomes between the four different combinations of the St. Gallen 2013 surrogate and PAM50 subtypes (Luminal B_SC_/Luminal B_PAM50_ [*n* = 64], Luminal B_SC_/Luminal A_PAM50_ [*n* = 90], Luminal A_SC_/Luminal B_PAM50_ [*n* = 5], and Luminal A_SC_/Luminal A_PAM50_ [*n* = 48]) is illustrated in Fig. 2e, f and Supplementary Fig. 2e, f. After 10 years of follow-up, patients with tumours classified as Luminal B_SC_/Luminal A_PAM50_, had better prognosis than those with tumours classified as Luminal B_SC_/Luminal B_PAM50_ (HR_BCFi_: 0.52, 95% CI: 0.33–0.83, *P* = 0.006; HR_OS_: 0.37, 95% CI: 0.21–0.66, *P* = 0.001; Table 2). A proportion (91/207) of the cohort was at risk for breast cancer events at 20 years of follow-up, hence the prognostic effects became weaker with long-term (>30 years) follow-up and the results were similar in multivariable analyses (Table 2).

### Predictive value of luminal PAM50 subtyping for tamoxifen benefit

**Fig. 3:**
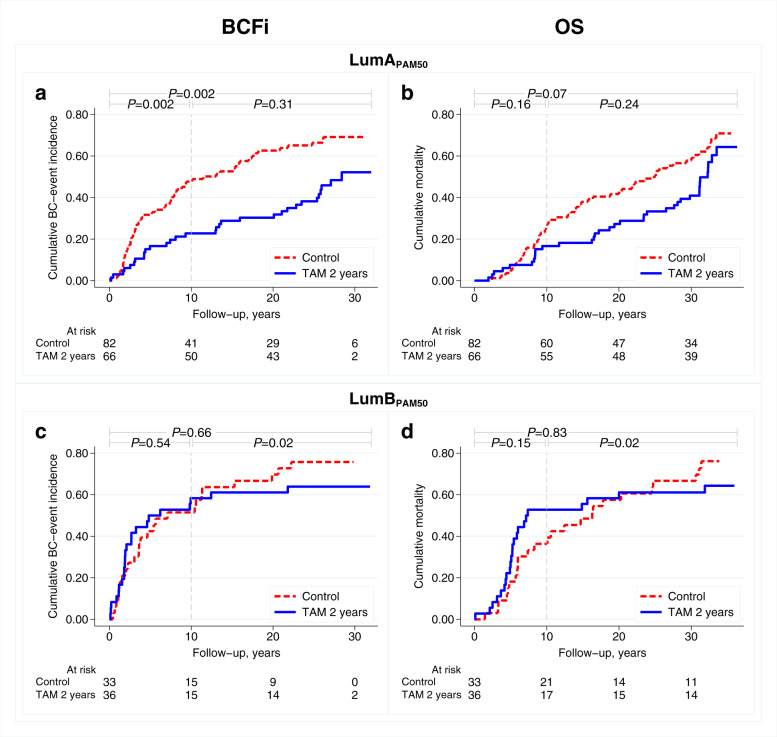
Cumulative incidence curves for BCFi and OS according to treatment arm. **(a**, **b)** Patients with LumA and (**c**, **d**) LumB tumours by PAM50. *P*-values from log rank test, Gehan’s version for BCFi, for maximum follow-up and for different time intervals. BCFi breast cancer-free interval, Lum Luminal, OS overall survival, TAM tamoxifen.

After 10 years of follow-up, a beneficial effect of adjuvant tamoxifen was observed in patients with ER+/HER2− and Luminal A_PAM50_ tumours (HR_BCFi_: 0.41, 95% CI: 0.23–0.74, *P* = 0.003; Fig. 3a); however, not for patients with Luminal B_PAM50_ tumours (HR_BCFi_: 1.19, 95% CI: 0.63–2.27, *P* = 0.59; Fig. 3c). Hence, the effect of tamoxifen was threefold better in patients with Luminal A_PAM50_ tumours as compared with those with Luminal B_PAM50_ tumours (interaction: HR_BCFi_: 0.34, 95% CI: 0.14–0.83, *P* = 0.02). Similar results were observed for OS (Table 4 and Fig. 3b, d) and in the multivariable analyses (Supplementary Table 2), but was not as evident after maximum follow-up. Corresponding cumulative incidence curves for RFi are presented in Supplementary Fig. 3. When selecting all patients with luminal PAM50 tumours, regardless of ER/HER2 status (*n* = 274), the findings after 10 years of follow-up were essentially the same (interaction: HR_BCFi_: 0.45, 95% CI: 0.21–0.96, *P* = 0.04)).

**Table 4:** Cox regression models for BCFi and OS by luminal PAM50 subtypes, tamoxifen treatment, and PAM50 subtype by treatment interaction in patients with ER-positive/HER2-negative tumours.

	BCFi	OS
	HR (95% CI); *P*-value	
	0–10 years	
	(*n* = 217, *n* = 92 events)	(*n* = 217, *n* = 64 events)
TAM vs control in LumA_PAM50_	0.41 (0.23–0.74); 0.003	0.61 (0.30–1.26); 0.18
TAM vs control in LumB_PAM50_	1.19 (0.63–2.27); 0.59	1.76 (0.85–3.63); 0.13
Interaction luminal PAM50 subtype x TAM	0.34 (0.14–0.83); 0.02	0.35 (0.13–0.97); 0.04
	>10 years^a^	
	(*n* = 121, *n* = 43 events)	(*n* = 153, *n* = 74 events)
TAM vs control in LumA_PAM50_	0.69 (0.35–1.37); 0.29	0.74 (0.44–1.25); 0.26
TAM vs control in LumB_PAM50_	0.17 (0.04–0.80); 0.03	0.25 (0.08–0.77); 0.02
Interaction luminal PAM50 subtype x TAM	4.05 (0.74–22.1); 0.11	2.95 (0.85–10.2); 0.09
	Maximum follow-up time^b^	
	(*n* = 217, *n* = 135 events)	(*n* = 217, *n* = 138 events)
TAM vs control in LumA_PAM50_	0.52 (0.34–0.81); 0.004	0.71 (0.46–1.08); 0.11
TAM vs control in LumB_PAM50_	0.80 (0.45–1.41); 0.44	0.87 (0.49–1.54); 0.63
Interaction luminal PAM50 subtype x TAM	0.65 (0.32–1.34); 0.24	0.82 (0.40–1.65); 0.57

All analyses are stratified by study region.

*BCFi*, breast cancer-free interval; *CI*, confidence interval; *ER*, oestrogen receptor; *HER2*, human epidermal growth factor receptor 2; *HR*, hazard ratio; Lum, Luminal; *OS*, overall survival; *TAM*, tamoxifen.

^a^From year 10 to maximum follow-up time.

^b^32 and 36 years regarding BCFi and OS, respectively.

### Prognostic value of ROR score (ER+/HER2− subgroup)

**Fig. 4:**
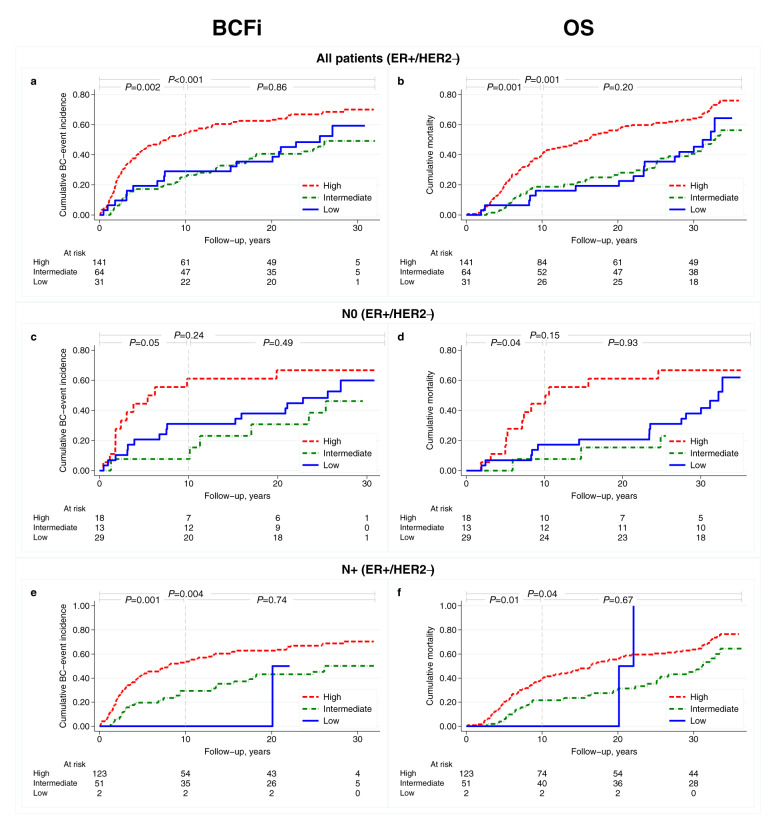
Cumulative incidence curves for BCFi and OS by ROR score categories for patients with ER-positive/HER2-negative tumors. (**a**, **b)** all patients (**c**, **d**) node-negative, and (**e**, **f**) node-positive patients. Overall *P*-values from log rank test, Gehan’s version for BCFi, for maximum follow-up and for different time intervals. BCFi breast cancer-free interval, ER oestrogen receptor, HER2 human epidermal growth factor receptor 2, OS overall survival, ROR risk of recurrence.

Among all patients (ER+/HER2− subgroup, *n* = 236), the distributions of low, intermediate, and high ROR score categories were: 13%, 27%, and 60%, respectively. The outcomes are illustrated in Fig. 4 and in Supplementary Fig. 4. For all patients with ER+/HER2− tumours, high vs low ROR score was associated with worse outcomes after 10 years of follow-up (HR_BCFi_: 2.36, 95% CI: 1.18–4.72, *P* = 0.02; Table 5). This effect was less pronounced after maximum long-term follow-up (HR_BCFi_: 1.70, 95% CI: 1.01–2.85, *P* = 0.04). The corresponding results for OS and the multivariable analyses are presented in Table 5 and in Supplementary Table 3, respectively.

**Table 5:** Cox regression univariable models for BCFi and OS by ROR score categories for different time intervals for patients with ER-positive/HER2-negative tumours in all patients, node-negative, and node-positive (1–3 positive nodes) subgroups of patients.

	All patients (ER+/HER2−)		Node-negative (ER+/HER2−)		Node-positive (ER+/HER2−)^a^	
	BCFi	OS	BCFi	OS	BCFi	OS
	HR (95% CI); P-value					
	0–10 years					
*ROR score*	(*n* = 236, *n* = 102 events) overall *P*-value < 0.001^d^	(*n* = 236, *n* = 74 events) overall *P*-value = 0.002^d^	(*n* = 60, *n* = 21 events) overall *P*-value = 0.01^d^	(*n* = 60, *n* = 14 events) overall *P*-value = 0.04^d^	(*n* = 121, *n* = 49 events)	(*n* = 121, *n* = 36 events)
Low (Ref.)	1.00	1.00	1.00	1.00	–	–
Intermediate	0.83 (0.37–1.88); 0.66	1.19 (0.42–3.37); 0.75	0.20 (0.03–1.59); 0.13	0.40 (0.05–3.43); 0.40	1.00	1.00
High	2.36 (1.18–4.72); 0.02	2.99 (1.19–7.46); 0.02	2.53 (1.04–6.12); 0.04	3.17 (1.03–9.75); 0.04	1.99 (1.08–3.66); 0.03	1.84 (0.91–3.74); 0.09
	>10 years^b^					
*ROR score*	(*n* = 130, *n* = 43 events) overall *P*-value = 0.93^d^	(*n* = 162, *n* = 77 events) overall *P*-value = 0.19^d^	(*n* = 39, *n* = 13 events) overall *P*-value = 0.56^d^	(*n* = 46, *n* = 16 events) overall P-value = 0.40^d^	(*n* = 69, *n* = 21 events)	(*n* = 84, n = 38 events)
Low (Ref.)	1.00	1.00	1.00	1.00	–	–
Intermediate	0.85 (0.36–2.01); 0.71	0.76 (0.37–1.56); 0.45	1.22 (0.38–3.92); 0.74	0.38 (0.08–1.75); 0.21	1.00	1.00
High	0.88 (0.39–2.01); 0.77	1.24 (0.65–2.35); 0.51	0.37 (0.05–3.03); 0.36	1.18 (0.36–3.79); 0.79	1.19 (0.50–2.80); 0.70	1.02 (0.54–1.93); 0.96
	Maximum follow-up^c^					
*ROR score*	(*n* = 236, *n* = 145 events) overall *P*-value = 0.001^d^	(*n* = 236, *n* = 151 events) overall *P*-value = 0.001^d^	(*n* = 60, *n* = 34 events) overall *P*-value = 0.15^d^	(*n* = 60, *n* = 30 events) overall *P*-value = 0.03^d^	(*n* = 121, *n* = 70 events)	(*n* = 121, *n* = 74 events)
Low (Ref.)	1.00	1.00	1.00	1.00	–	–
Intermediate	0.84 (0.47–1.53); 0.58	0.89 (0.49–1.61); 0.70	0.66 (0.26–1.69); 0.39	0.39 (0.11–1.35); 0.14	1.00	1.00
High	1.70 (1.01–2.85); 0.04	1.77 (1.06–2.97); 0.03	1.69 (0.79–3.58); 0.17	1.96 (0.91–4.22); 0.09	1.68 (1.03–2.75); 0.04	1.34 (0.84–2.14); 0.22

All analyses are stratified by study region. The ROR score categories are defined by the following cut-offs based on N-status; N0; low: 0–40, intermediate: 41–60, high: 61–100, N1; low: 0–15, intermediate: 16–40, high: 41–100, N2: high: 0–100.

*BCFi* breast cancer-free interval, *CI* confidence interval, *ER* oestrogen receptor, *HER2* human epidermal growth factor receptor 2, *HR* hazard ratio, *OS* overall survival, *ROR* risk of recurrence.

^a^Only N1 (1–3 positive nodes) are included in the node-positive definition. Since only n = 2 patients are defined as ROR low in the N1 category, these are omitted from the analyses.

^b^From year 10 to maximum follow-up time.

^c^32 and 36 years regarding BCFi and OS, respectively.

^d^Two-degree of freedom Wald test.

Stratified by nodal status, the distributions of ROR score categories were: node-negative (N0) (*n* = 60): 48%, 22% and 30%, N+ (1–3 positive nodes, *n* = 123): 2%, 42% and 57%, respectively. High vs low ROR score yielded 2.5- and 1.7- fold increased incidence of breast cancer events in N0 patients after 10 years of follow-up and maximum follow-up, respectively (Table 5). Due to small sample size (*n* = 2), the low ROR category was omitted in the analysis of N+ (1–3 positive nodes) patients and the results indicated that intermediate ROR score was associated with lower incidence of breast cancer events than high ROR score (Table 5).

## Discussion

This study demonstrates that PAM50 subtypes and ROR score could provide long-term prognostic information in premenopausal patients, and indicates a possible tamoxifen-predictive effect by luminal intrinsic subtyping after 10 years of follow-up. The Prosigna^©^ Breast Cancer Prognostic Gene Signature Assay, which can use formalin-fixed paraffin-embedded tissues in a decentralised mode^20^, is validated in postmenopausal women^7,8^. Our results demonstrated that premenopausal patients with Luminal B_PAM50_ as compared with Luminal A_PAM50_ tumours, had a > 1.4-fold higher incidence of breast cancer events and death after long-term follow-up.

Our results confirmed the suboptimal agreement between luminal intrinsic and surrogate subtyping^16–19^, and we demonstrated that over 50% of Luminal B_SC_ tumours were re-classified as Luminal A_PAM50._ These results are in agreement with the report by Viale et al. using BluePrint/MammaPrint^17^. Importantly, the re-classification in our study was translated into improved prognosis and this emphasises the possibility of overestimating the risk for breast cancer events in patients with surrogate Luminal B tumours, which could affect treatment decisions.

Two years of adjuvant tamoxifen was previously reported to be associated with a long-term survival benefit and reduction of breast cancer events for ER-positive patients by the SBII:2pre trial^21,22^. In this study, during the first 10 years of follow-up, a tamoxifen-treatment effect was seen in patients with Luminal A_PAM50_ tumours, but not in those with Luminal B_PAM50_ tumours. However, the evidence of treatment interaction was weak in the subsequent follow-up time periods. This indicated that the luminal PAM50 subtype could be a predictive marker for tamoxifen benefit in addition to ER status. Our finding was observed regardless of ER and HER2 status by IHC/ISH, suggesting the PAM50 subtyping could potentially be clinically used upfront to stratify premenopausal patients to tamoxifen therapy. The luminal PAM50 subtypes have previously been reported to be associated with benefit from 5 years adjuvant tamoxifen treatment in premenopausal women (*n* = 398); however, no separate treatment effects in patients with Luminal A and B were reported^12^. Yu et al. presented a long-term beneficial effect of 5-year tamoxifen treatment in postmenopausal women with Luminal A_PAM50_ tumours, and the effect attenuated over time in patients with Luminal B_PAM50_ tumours^23^.

This study demonstrated better long-term prognosis for patients with low vs high ROR score. However, the trial was underpowered to define the prognostic value of ROR score by nodal status. In general, our cohort had more aggressive tumour characteristics and the outcome for node-negative patients with low ROR score was worse compared with postmenopausal women in the validation studies^7,8,20^. It remains unclear if premenopausal patients with low ROR score are potential candidates for abstaining adjuvant chemotherapy. Data from the TAILORx trial indicated a beneficial effect of adjuvant chemotherapy in patients ≤50 years and a recurrence score of 16–25^24^ and the results from the RxPONDER demonstrated that node-positive premenopausal women with a recurrence score ≤25 did benefit from additional chemotherapy^25^. These results are emphasising that further studies of multigene assays including premenopausal patients are warranted.

A strength of this study is the long-term follow-up data and the fact that it is based on a trial including only premenopausal women randomised to tamoxifen monotherapy vs control (systemically untreated patients). Moreover, reassessments of the progesterone receptor and Ki67 were performed for the surrogate subtypes and a well-established gene expression method was used. A limitation of this study is that the quality of old preserved tissues may result in uncertainty, especially regarding Ki67 assessment^26^. However, the required RNA quantity is minimal, and quality check of the RNA assured that gene expression output data were reliable. Further limitations include that the duration of endocrine treatment was shorter than current recommendation and, the power of this study was low due to the limited number of included patients. Even though we demonstrated that PAM50 subtyping and ROR score could separate premenopausal into groups with different risks of recurrence and death, it remains unclear if these can be used for de-escalation of adjuvant chemotherapy^27^.

In conclusion, PAM50 subtypes and ROR score provided independent prognostic information after long-term follow-up. After 10 years of follow-up, the re-classification of Luminal B_SC_ tumours into Luminal A_PAM50_ was associated with a lower incidence of breast cancer events. Moreover, the tamoxifen effect was associated with the Luminal A intrinsic subtype, independent of ER status.

## Methods

### Study population

The patients in this study were included in the SBII:2pre study, which randomised 564 premenopausal women between 2 years of adjuvant tamoxifen or no systemic treatment. Inclusion and exclusion criteria have been described previously and demonstrated long-term beneficial effect of tamoxifen treatment^21,22,28,29^. Patients were classified as premenopausal until one year after menstrual periods had stopped according to the study protocol (Supplementary Reference 1). The cohort included in this study is illustrated in Fig. 1.

### Compliance with ethical requirements

Oral informed consent was obtained from all participants included in the SBII:2pre trial, and approval was given by the ethical committees in Lund and Linköping, Sweden. The oral consent was verified by a signature of the investigator in the registration form which was sent to the coordinating centre. The follow-up study was approved by the ethical committee of Lund (Dnr LU 2015/350) for extended follow-up as well as for genomic analysis (Dnr LU 2017/97). Biobank approval was cleared for all involved pathology departments.

### Study endpoints and follow-up data

The primary endpoints were BCFi including any of the following first events: local, regional, or distant recurrence; contralateral breast cancer (invasive or ductal cancer in situ); or breast cancer-related death (data cut-off Nov 30 2016)^22^, according to the DATECAN recommendation^30^. The secondary endpoint was OS and follow-up data were retrieved from the Swedish Causes of Death Register (data cut-off 10 December 2020). In sensitivity analysis, we additionally reported on RFi excluding contralateral breast cancer events^30^. Results for maximum follow-up and the two time intervals 0–10 years and >10 years were reported.

### Tumour characteristics and assessments of progesterone receptor (PR) and Ki67 status

Archival formalin-fixed paraffin-embedded (FFPE) tissues (*n* = 520) from breast tumours of the study participants were collected. Reassessments of PR (*n* = 464) and Ki67 (*n* = 463) status using whole tissue sections were performed according to Swedish national guidelines by a breast pathologist (UK)^31^. Data on ER, Nottingham histological grade (NHG), and HER2 was available as described previously^21,28,29,32,33^. These assessments were performed retrospectively and independently by two national reference breast pathologists^28,33^.

### Gene expression analyses

1–5 sections (10 µm thick) from FFPE tissue with invasive breast carcinoma, were used to extract RNA (AllPrep DNA/RNA FFPE kit (Qiagen Cat:80234, Hilden, Germany)). Gene expression analysis was performed according to the manufacturer’s instructions using the NanoString Breast Cancer 360^TM^ assay on an nCounter^®^ SPRINT Profiler instrument (NanoString Technologies)^34^.

Housekeeping gene geomean quality control (QC) categorised samples as PASS/BORDERLINE (≥202) or FAIL (<202) with 91% (437/479) of the samples passing QC. PAM50 genes were normalised to the PAM50 housekeeper gene geomean. The correlation between the observed scaled expression for the PAM50 genes and a centroid for each of the four subtypes was then determined. The subtype with the greatest correlation value defined the intrinsic subtype. In the ROR score, a weighted sum of the proliferation score, the four subtype correlations and tumour size were used to calculate a score between 0 and 100. The categorisation of ROR score was determined based on nodal status according to the following definitions: N0 low: 0–40, intermediate: 41–60, high: 61–100, N1 (1–3 positive nodes); low: 0–15, intermediate: 16–40, high: 41–100, N2 (≥4 positive nodes); high: 0–100^20^.

### Surrogate subtyping

The ER+/HER2− breast cancer tumours were classified as Luminal A_SC_ or Luminal B_SC_ according to the St. Gallen 2013 guidelines: Luminal A_SC_; low Ki67 (<20%) and high PR (≥20%), Luminal B_SC_; high Ki67 (≥20%) and/or low PR (<20%)^35^.

### Statistical analyses

Cumulative incidence curves were used to illustrate outcomes for patient subgroups. The estimates for BCFi take the competing event death without a preceding breast cancer event into account. In analyses of RFi, also contralateral breast cancer as first event was treated as a competing risk. Evidence against equality of two or more cumulative incidence curves was evaluated using the log rank test. We used a trend version of the test for ordered groups and a modified version, derived by Geskus^36^, for comparison of cause-specific cumulative incidence curves (BCFi and RFi). Cox regression models, stratified for region, were used to estimate HRs with 95% CIs Cause-specific Cox regression was used for the endpoint BCFi, censoring the follow-up at time of death for patients who died without a registered breast cancer event. Similarly, the follow-up time was censored at the time of death without a preceding breast cancer event or at the diagnosis of contralateral breast cancer as first breast cancer related event in analyses of RFi. Proportional hazards assumptions were in general not met in analyses of long-term follow-up. The corresponding HRs should therefore be interpreted cautiously as average effects over time. Our way of handling this problem was to also calculate the relative effects with the follow-up restricted to 10 years. The evidence against proportional hazards, as measured by Schoenfeld’s test, was in general much lower for the two intervals 0–10 years and 10+ years compared to the evidence in analyses of maximum follow-up.

Log rank tests of prognostic or predictive effects are presented in figures whereas tests based on Cox regression models are presented in tables. For Cox models, we present both effects relative to a chosen reference category, with 95% CIs and *P*-values, and an overall Wald test of each factor. Multivariable analyses of PAM50 subtype and ROR categories were adjusted for established prognostic factors, but since nodal status and tumour stage are included in the definition of ROR, these factors were excluded from the set of adjustment variables in analyses of the independent prognostic effect of ROR categories.

For prognostic differences between luminal PAM50 and surrogate subtyping, a variable including the four combinations of Luminal A_PAM50_, Luminal B_PAM50_, Luminal A_SC_ and Luminal B_SC_ tumours, was created. Percentage agreement and kappa (κ) statistics were used in agreement analyses. To evaluate the differential effect of tamoxifen benefit in luminal PAM50 tumours, a Cox model was fitted including an interaction variable between luminal PAM50 subgroup and treatment arm. The prognostic effect of ROR score was studied in ER+/HER2− patients and also stratified by nodal status.

The results are presented in accordance with the Reporting Recommendations for Tumour Marker Prognostic Studies (REMARK) where applicable^37,38^. All statistical tests were two-sided, and a *P*-value <0.05 was considered statistically significant. No adjustment for multiple testing was performed. All calculations were performed using IBM SPSS, version 25.0 (IBM Corp., Armonk, NY, USA) and the cumulative incidence curves were drawn using STATA, version 17.0 (StataCorp LLC, College Station, TX, USA).

### Reporting summary

Further information on research design is available in the Nature Research Reporting Summary linked to this article.

## Supplementary information

Supplementary information

Reporting Summ﻿ary Checklist

## Data Availability

The datasets used and/or analysed during the current study could be available from the corresponding author upon reasonable request if this is in line with current laws.

## Code availability

No code was generated in the present study. Assignment of PAM50 and ROR score, were provided by the manufacturer’s code (NanoString Breast Cancer 360^TM^ assay on an nCounter^®^ SPRINT Profiler instrument, NanoString Technologies)^34^.
