# Supplementary material for: PAM50 subtyping and ROR score add long-term prognostic information in premenopausal breast cancer patients
Source: NPJ Breast Cancer. 2022 May 9;8:61. doi: 10.1038/s41523-022-00423-z (PMC9085780; doi:10.1038/s41523-022-00423-z)
Supplement: Supplementary file 1 — Supplementary information [file 41523_2022_423_MOESM1_ESM.pdf]

Supplementary Table 1. Cox regression uni- and multivariable models for BCFi and OS by PAM50 subtypes and St. Gallen 2013 surrogate subtypes in all patients and for different time intervals

	Univariable		Multivariable[a]	
	BCFi	OS	BCFi	OS
	HR (95% CI); P-value			
PAM50 intrinsic subtype (all patients)	0–10 years			
	(n=437, n=218 events) overall P-value<0.001[d]	(n=437, n=176 events) overall P-value<0.001[d]	(n=411, n=203 events) overall P-value=0.01[d]	(n=411, n=166 events) overall P-value<0.001[d]
LumA (Ref.)	1.00	1.00	1.00	1.00
LumB	1.88 (1.31–2.71); 0.001	2.33 (1.51–3.58); <0.001	1.78 (1.18–2.68); 0.006	2.08 (1.28–3.38); 0.003
HER2-E	2.55 (1.77–3.69); <0.001	4.08 (2.70–6.18); <0.001	1.99 (1.23–3.24); 0.005	2.94 (1.71–5.03); <0.001
Basal-like	2.09 (1.45–2.99); <0.001	3.36 (2.23–5.06); <0.001	1.95 (1.19–3.22); 0.008	3.00 (1.73–5.22); <0.001
	>10 years[b]			
	(n=210, n=68 events) overall P-value=0.53[d]	(n=261, n=124 events) overall P-value=0.41[d]	(n=200, n=66 events) overall P-value=0.14[d]	(n=245, n=119 events) overall P-value=0.41[d]
LumA (Ref.)	1.00	1.00	1.00	1.00
LumB	1.05 (0.55–2.00); 0.89	0.85 (0.52–1.39), 0.50	1.92 (0.92–4.01); 0.08	1.16 (0.66–2.04); 0.62
HER2-E	0.50 (0.20–1.29); 0.15	0.70 (0.38–1.29); 0.25	1.27 (0.33–4.90): 0.73	1.77 (0.76–4.13); 0.19
Basal-like	0.95 (0.50–1.82); 0.88	0.69 (0.40–1.17); 0.17	2.58 (0.94–7.08); 0.07	1.90 (0.86–4.18); 0.11
	Maximum follow-up time[c]			
	(n=437, n=286 events) overall P-value<0.001[d]	(n=437, n=300 events) overall P-value<0.001[d]	(n=411, n=269 events) overall P-value=0.009[d]	(n=411, n=285 events) overall P-value=0.001[d]
LumA (Ref.)	1.00	1.00	1.00	1.00
LumB	1.60 (1.16–2.18); 0.004	1.41 (1.03–1.93); 0.03	1.66 (1.17–2.37), 0.005	1.48 (1.04–2.10); 0.03
HER2-E	1.84 (1.32–2.56); <0.001	1.99 (1.45–2.73); <0.001	1.81 (1.16–2.81); 0.009	2.19 (1.43–3.36); <0.001
Basal-like	1.69 (1.24–2.31), 0.001	1.68 (1.24–2.28); 0.001	1.92 (1.24–2.99); 0.004	2.24 (1.45–3.45); <0.001

[a]All analyses are stratified by study region and adjusted for age (continuous), tumour size (>20 vs ≤20mm), NHG (1 vs 2 vs 3), nodal status (N0 vs N1 vs N2) and treatment arm in addition to surrogate/molecular subtype
[b]From year 10 to maximum follow-up time
[c]32 and 36 years regarding BCFi and OS, respectively
[d]Three degree of freedom Wald test
Abbreviations: BCFi, breast cancer-free interval; CI, confidence interval; HER2-E, human epidermal growth factor receptor 2-enriched; HR, hazard ratio; Lum, Luminal; NHG, Nottingham histological grade; OS, overall survival; SC, surrogate classification

Supplementary Table 2. Cox regression multivariable models for for BCFi and OS by luminal PAM50 subtypes, tamoxifen treatment and PAM50 subtype by treatment interaction in patients with ER-positive/HER2-negative tumours

	BCFi	OS
	HR (95% CI); P-value	
	0–10 years	
	(n=214, n=92 events)	(n=214, n=64 events)
TAM vs control in $LumA_{PAM50}$	0.42 (0.23–0.77); 0.005	0.68 (0.33–1.41); 0.30
TAM vs control in $LumB_{PAM50}$	1.11 (0.58–2.14); 0.75	1.69 (0.81–3.54); 0.16
Interaction luminal PAM50 subtype x TAM	0.38 (0.16–0.92); 0.03	0.40 (0.14–1.14); 0.09
	>10 years[a]	
	(n=118, n=42 events)	(n=150, n=74 events)
TAM vs control in $LumA_{PAM50}$	0.73 (0.36–1.49); 0.39	0.68 (0.40–1.15); 0.15
TAM vs control in $LumB_{PAM50}$	0.15 (0.03–0.73); 0.02	0.19 (0.06–0.61); 0.005
Interaction luminal PAM50 subtype x TAM	4.88 (0.86–27.6); 0.07	3.48 (0.99–12.2); 0.05
	Maximum follow-up time[b]	
	(n=214, n=134 events)	(n=214, n=138 events)
TAM vs control in $LumA_{PAM50}$	0.54 (0.35–0.85); 0.007	0.74 (0.48–1.13); 0.16
TAM vs control in $LumB_{PAM50}$	0.77 (0.43–1.38); 0.38	0.81 (0.45–1.45); 0.48
Interaction luminal PAM50 subtype x TAM	0.71 (0.34–1.47); 0.35	0.90 (0.44–1.84); 0.77

All analyses were stratified by study region and adjusted for age (continuous), nodal status (N0 vs N1 vs N2), tumour size (>20 vs ≤20mm), NHG (1 vs 2 vs 3) in addition to treatment arm.

[a]From year 10 to maximum follow-up time

[b]32 and 36 years regarding BCFi and OS, respectively

Abbreviations: BCFi, breast cancer-free interval; CI, confidence interval; ER, oestrogen receptor; HER2, human epidermal growth factor receptor 2; HR, hazard ratio; Lum, Luminal; NHG, Nottingham histological grade; OS, overall survival; TAM, tamoxifen

Supplementary Table 3. Cox regression multivariable models for BCFi and OS by ROR score categories for different time intervals for patients with ER-positive/HER2-negative tumours in all patients, node-negative, and node-positive (1–3 positive nodes) patients

	All patients		Node-negative		Node-positive[a]	
	BCFi	OS	BCFi	OS	BCFi	OS
	HR (95% CI); P-value					
	0–10 years					
ROR score	(n=233, n=102 events) overall P-value<0.001[d]	(n=233, n=74 events) overall P-value=0.04[d]	(n=59, n=21 events) overall P-value=0.06[d]	(n=59, n=14 events) overall P-value=0.33[d]	(n=119, n=49 events)	(n=119, n=36 events)
Low (Ref.)	1.00	1.00	1.00	1.00	-	-
Intermediate	0.82 (0.36–1.85); 0.63	1.20 (0.42–3.42); 0.73	0.20 (0.02–1.60); 0.13	0.18 (0.02–1.94); 0.16	1.00	1.00
High	2.20 (1.09–4.47); 0.03	2.38 (0.93–6.07); 0.07	2.47 (0.68–8.94); 0.17	0.73 (0.09–6.02); 0.77	1.85 (0.95–3.58); 0.07	1.32 (0.61–2.88); 0.48
	>10 years[b]					
ROR score	(n=127, n=42 events) overall P-value=0.62[d]	(n=159, n=77 events) overall P-value=0.01[d]	(n=38, n=12 events)	(n=45, n=16 events)	(n=67, n=21 events)	(n=82, n=38 events)
Low (Ref.)	1.00	1.00	1.00	1.00	-	-
Intermediate	1.07 (0.43–2.67); 0.88	0.78 (0.38–1.60); 0.49	2.39 (0.64–8.94); 0.20	0.63 (0.13–3.08); 0.56	1.00	1.00
High	1.44 (0.59–3.48); 0.42	1.74 (0.90–3.39); 0.10	-	-	1.13 (0.43–2.95); 0.81	1.29 (0.66–2.53); 0.46
	Maximum follow-up[c]					
ROR score	(n=233, n=144 events) overall P-value=0.001[d]	(n=233, n=151 events) overall P-value=0.001[d]	(n=59, n=33 events) overall P-value=0.05[d]	(n=59, n=30 events) overall P-value=0.02[d]	(n=119, n=70 events)	(n=119, n=74 events)
Low (Ref.)	1.00	1.00	1.00	1.00	-	-
Intermediate	0.91 (0.50–1.67); 0.77	0.88 (0.49–1.59); 0.68	0.78 (0.30–2.07); 0.62	0.39 (0.11–1.40); 0.15	1.00	1.00
High	1.88 (1.09–3.24); 0.02	1.79 (1.05–3.04), 0.03	3.40 (1.10–10.52); 0.03	3.33 (1.03–10.73); 0.04	1.57 (0.92–2.68); 0.10	1.26 (0.76–2.09); 0.37

All analyses are stratified by study region and adjusted for age (continuous variable), NHG (1 vs 2 vs 3) and treatment arm.
The ROR score categories were defined by the following cut-offs based on N-status; N0; low: 0–40, intermediate: 41–60, high: 61–100, N1; low: 0–15, intermediate: 16–40, high: 41–100, N2; high: 0–100.
[a]Only N1 (1–3 positive nodes) included in the node-positive definition. Since only n=2 patients were defined as ROR low in the N1 category, these were omitted from the analyses
[b]From year 10 to maximum follow-up time
[c]32 and 36 years regarding BCFi and OS, respectively
[d]Two degree of freedom Wald test
Abbreviations: BCFi, breast cancer-free interval; CI, confidence interval; ER, oestrogen receptor; HER2, human epidermal growth factor receptor 2; HR, hazard ratio; NHG, Nottingham histological grade; OS, overall survival; ROR, Risk of Recurrence

SUPPLEMENTARY FIGURE LEGENDS

Supplementary Fig. 1 Distribution of PAM50 subtypes
(a) all (*n*=437), (b) node-negative (*n*=119), and (c) node-positive (*n*=316) patients. In total, *n*=2 cases with missing nodal status

Abbreviations: HER2-E, human epidermal growth factor receptor 2-enriched; Lum, Luminal

Supplementary Fig. 2 Cumulative incidence curves for RFi and OS by PAM50 subtypes
(a–b) all included patients, (c–d) patients with ER-positive/HER2-negative tumours, and (e-f) patients with available intrinsic PAM50 and surrogate subtyping by St. Gallen 2013

Overall *P*-values from log rank test, Gehan's version for RFi, for maximum follow-up and for different time intervals.

Abbreviations: ER, oestrogen receptor; HER2-E, human epidermal growth factor receptor 2-enriched; Lum, Luminal; OS, overall survival; RFi, recurrence-free interval; SC, surrogate classification

Supplementary Fig. 3 Cumulative incidence curves for RFi and OS according to treatment arm
(a-b) patients with LumA and (c–d) LumB tumours by PAM50

P-values from log rank test, Gehan's version for RFi, for maximum follow-up and for different time intervals.

Abbreviations: Lum, Luminal; OS, overall survival; RFi, recurrence-free interval; TAM, tamoxifen

Supplementary Fig. 4 Cumulative incidence curves for RFi and OS by ROR score categories for patients with ER-positive/HER2-negative tumours
(a–b) all patients (c–d) node-negative, and (e–f) node-positive patients

Overall *P*-values from log rank test, Gehan's version for RFi, for maximum follow-up and for different time intervals.

Abbreviations: ER, oestrogen receptor; HER2, human epidermal growth factor receptor 2; OS, overall survival; RFi, recurrence-free interval; ROR, risk of recurrence; TAM, tamoxifen

Supplementary Fig. 1

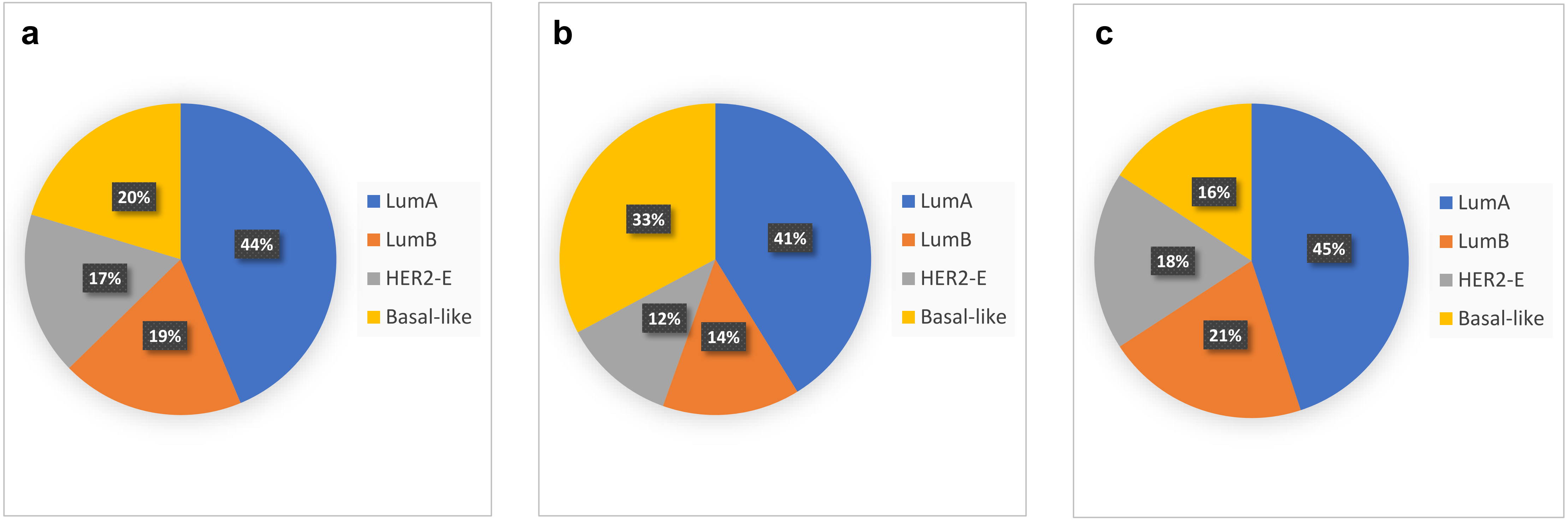

Supplementary Fig. 2

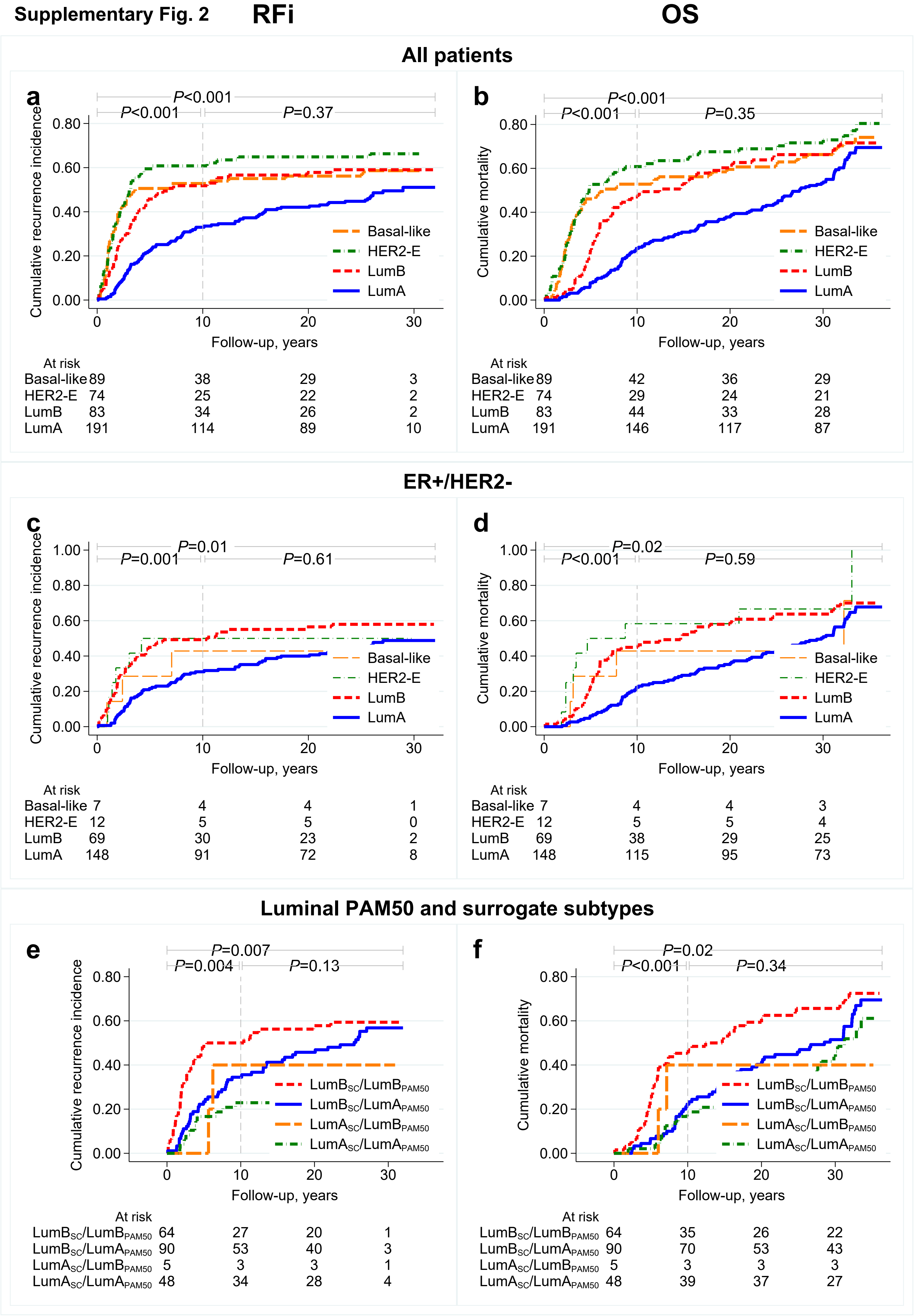

Supplementary Fig. 3

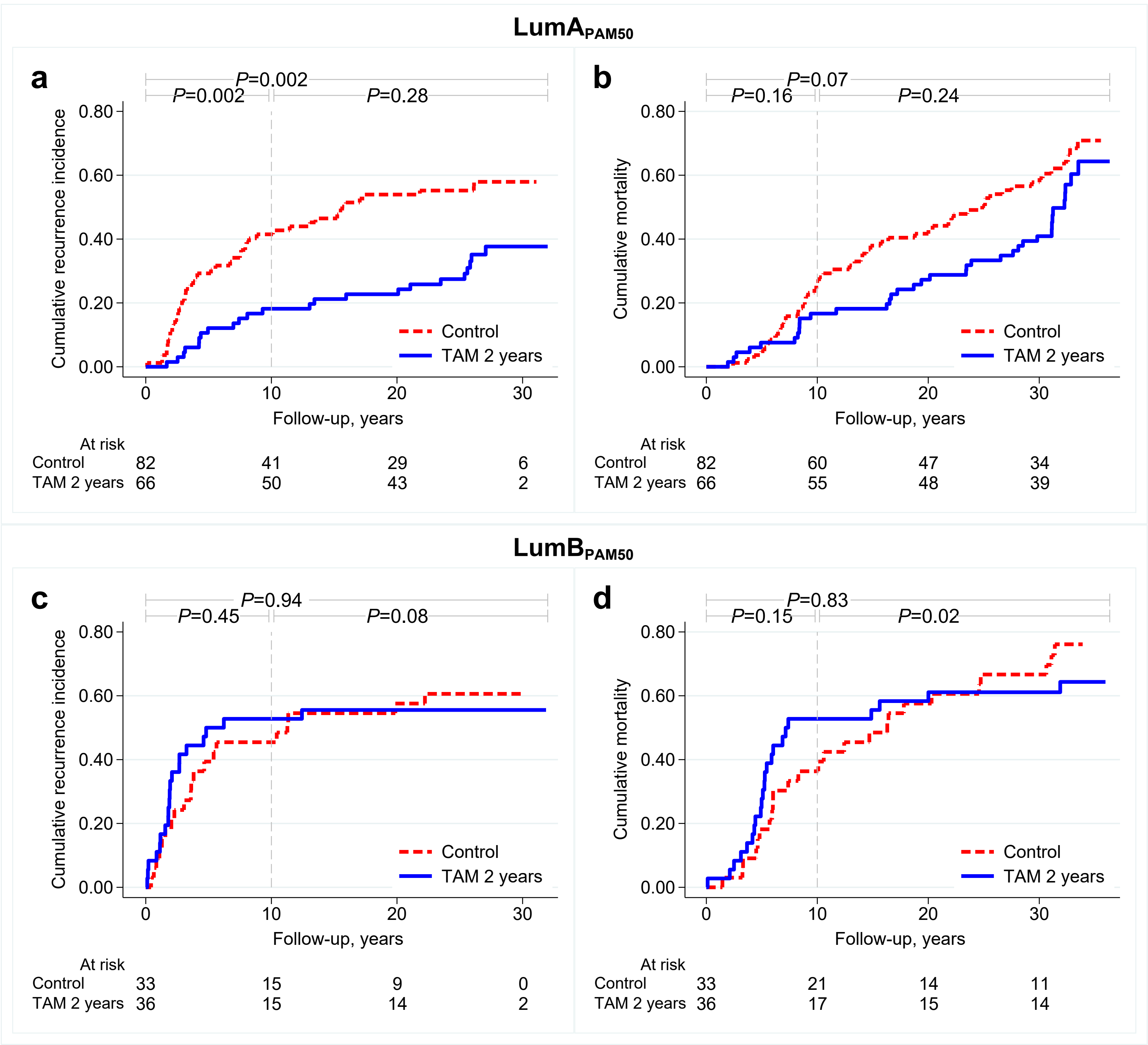

Supplementary Fig. 4

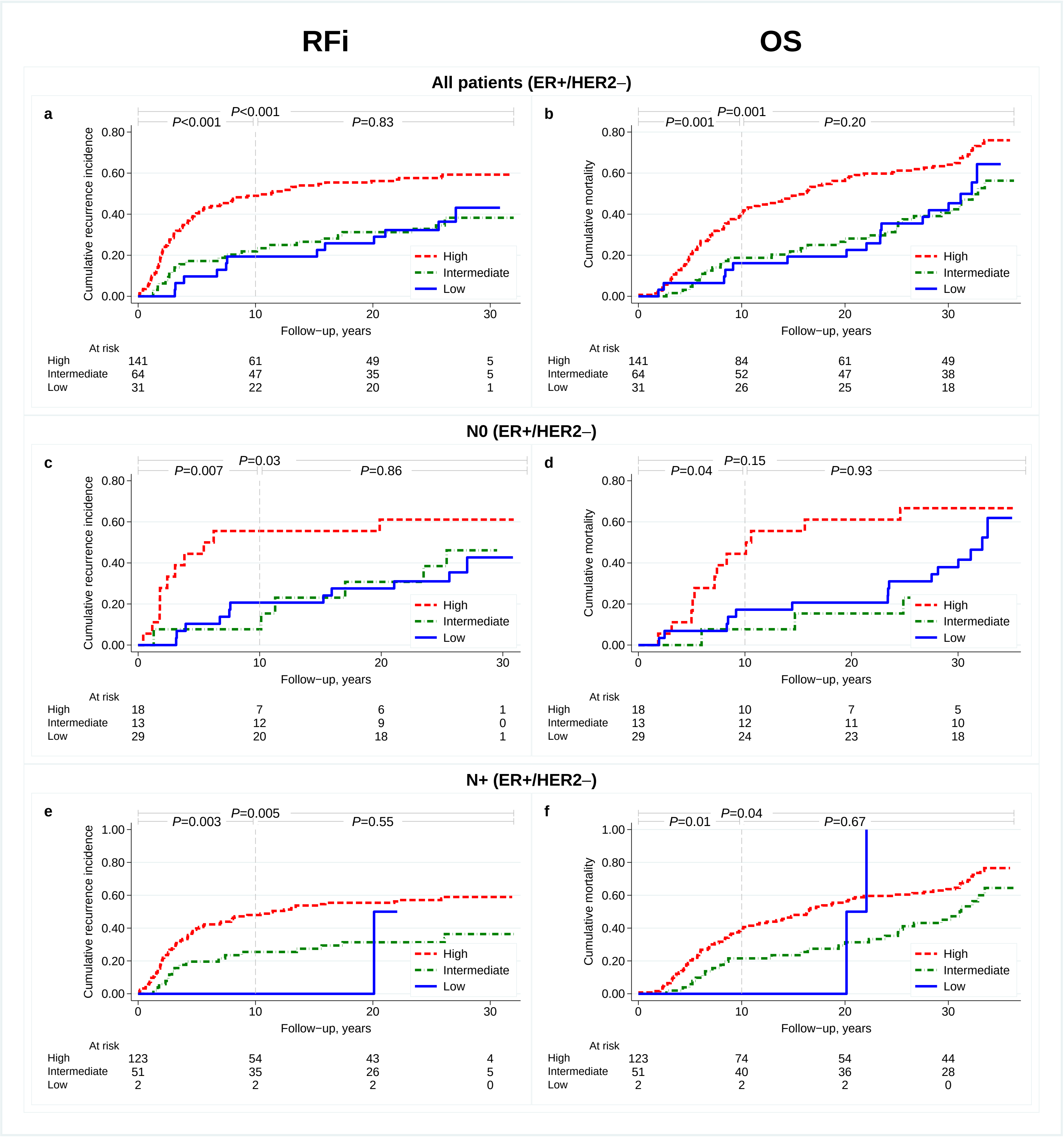

Supplementary Reference 1. Abbreviated translated version of the study protocol

TRANSLATION OF THE SWEDISH PROTOCOL FOR THE SBII:2PRE TRIAL

This protocol also includes the SBII:2post Trial for postmenopausal women and the text regarding that trial has not been translated. There was also a plan for a Radiotherapy Trial, which was never conducted.

One should keep in mind that the SBII:2pre Trial was planned more than 30 years ago in accordance with the contemporary clinical practice.

The current follow-up includes overall mortality and breast cancer-related mortality and the methods and statistics regarding this is described in the submitted manuscript.

SELECTION OF PATIENTS, INCLUDING BOTH ELIGIBILITY AND INELIGIBILITY CRITERIA

Inclusion critera

- The patient should have been diagnosed with infiltrating breast cancer
- The patient should be premenopausal, defined as less than 1 year since the last menstruation
- The patient should have undergone modified radical mastectomy or breast conserving therapy
- The patient should have undergone radical surgery
- The patient should not have distant metastasis.

Exclusion criteria

- Postmenopausal patients, defined as more than 1 year since the last menstruation
- Patients with ongoing pregnancy or lactation
- Patients with breast cancer stage 0, I, III, or IV
- Bilateral breast cancer
- Patients previously treated for malignant tumor disease, except for basal cell carcinoma, cervix cancer and endometrial cancer stage 0
- Patient not expected to cooperate to the extent that the treatment and follow-up require
- Patients that decline to participate

Stratification and randomization

- Randomization will be performed for patients that fulfill the inclusion criteria's and lack all exclusion criteria.
- The patients will be stratified according to the hospital responsible for the surgery.
- The randomization will be performed through telephone contact to the Regional Tumor Register, the University hospital of Lund, Monday to Friday.
- Randomization will be performed in blocks by 8.

SCHEMA AND TREATMENT PLAN, INCLUDING ADMINISTRATION SCHEDULE

- Modified radical surgery according to appendix yy (not available)
- Postoperative radiotherapy according to appendix (not available)

- **Medical treatment**
  - Tamoxifen 20 or 40 mg orally daily for 2 years
  - Control (no adjuvant endocrine therapy)

RULES FOR DOSE MODIFICATION

The trial was performed by two cooperating trial centers. Patients recruited by the South Swedish oncology center (n=427) received 20 mg of tamoxifen while the patients recruited by the South Eastern center (n=137) received 40 mg. (The optimal dose was not known at the time of the trial.)

MEASUREMENT OF TREATMENT PLAN INCLUDING RESPONSE CRITERIA, DEFINITIONS OF RESPONSE AND SURVIVAL, AND METHODS OF MEASUREMENTS

Follow-up

The patients will have follow-up:

- Clinical examination should be performed every 6 month for 5 years
- Chest X-ray and mammogram should be performed annually during 10 years

Evaluation

- Recurrence-free survival
- Site of recurrence
- Toxicity
- Survival

End of treatment

- Patients randomized to tamoxifen should end treatment after 2 years from the start of the treatment.
- The participating centers are notified 2 month before the planned discontinuation of the drug.

REASONS FOR EARLY CESSATION OF THERAPY

- The tamoxifen treatment should be stopped in case of recurrence. It should also be stopped if the patient does not tolerate the drug or if the patient chooses not to continue with the treatment. In all these cases the study secretariat should be informed.
- Patients who stop the treatment without signs of recurrence should continue the follow-up in the same way as those who fulfill the treatment.

OBJECTIVES AND ENTIRE STATISTICAL SECTION

- The study is planned, with a 90% power and an alpha-level of 5%, to detect an absolute difference of 15% regarding 5-year recurrence free survival.
- If in one of the two arms side effects, recurrences or mortality is increased, this group will be discontinued.
- Decisions regarding discontinuation of the study will be taken together by all involved parties.
