# Supplementary material for: PAM50 subtyping and ROR score add long-term prognostic information in premenopausal breast cancer patients
Source: NPJ Breast Cancer. 2022 May 9;8:61. doi: 10.1038/s41523-022-00423-z (PMC9085780; doi:10.1038/s41523-022-00423-z)
Supplement: Supplementary file 2 — Reporting Summ﻿ary Checklist [file 41523_2022_423_MOESM2_ESM.pdf]

nature portfolio

Corresponding author(s): Christine Lundgren

Last updated by author(s): Feb 7, 2022

Reporting Summary

Nature Portfolio wishes to improve the reproducibility of the work that we publish. This form provides structure for consistency and transparency in reporting. For further information on Nature Portfolio policies, see our Editorial Policies and the Editorial Policy Checklist.

Statistics

For all statistical analyses, confirm that the following items are present in the figure legend, table legend, main text, or Methods section.

n/a	Confirmed	
☐	☒	The exact sample size (n) for each experimental group/condition, given as a discrete number and unit of measurement
☒	☐	A statement on whether measurements were taken from distinct samples or whether the same sample was measured repeatedly
☐	☒	The statistical test(s) used AND whether they are one- or two-sided<br>*Only common tests should be described solely by name; describe more complex techniques in the Methods section.*
☐	☒	A description of all covariates tested
☐	☒	A description of any assumptions or corrections, such as tests of normality and adjustment for multiple comparisons
☐	☒	A full description of the statistical parameters including central tendency (e.g. means) or other basic estimates (e.g. regression coefficient) AND variation (e.g. standard deviation) or associated estimates of uncertainty (e.g. confidence intervals)
☐	☒	For null hypothesis testing, the test statistic (e.g. F, t, r) with confidence intervals, effect sizes, degrees of freedom and P value noted<br>*Give P values as exact values whenever suitable.*
☒	☐	For Bayesian analysis, information on the choice of priors and Markov chain Monte Carlo settings
☒	☐	For hierarchical and complex designs, identification of the appropriate level for tests and full reporting of outcomes
☐	☒	Estimates of effect sizes (e.g. Cohen's d, Pearson's r), indicating how they were calculated

Our web collection on statistics for biologists contains articles on many of the points above.

Software and code

Policy information about availability of computer code

Data collection	Clinical data and outcome data were retrieved from initial CRFs, patient´s charts and central registries. RNA were extracted from archived tissues (AllPrep DNA/RNA FFPE kit (Qiagen Cat:80234, Hilden, Germany).
Data analysis	Statistical analyses were carried in SPSS and STATA. Gene expression analyses were performed according to the manufacturer's instructions using the NanoString Breast Cancer 360TM assay .

For manuscripts utilizing custom algorithms or software that are central to the research but not yet described in published literature, software must be made available to editors and reviewers. We strongly encourage code deposition in a community repository (e.g. GitHub). See the Nature Portfolio guidelines for submitting code & software for further information.

Data

Policy information about availability of data

All manuscripts must include a data availability statement. This statement should provide the following information, where applicable:

- Accession codes, unique identifiers, or web links for publicly available datasets
- A description of any restrictions on data availability
- For clinical datasets or third party data, please ensure that the statement adheres to our policy

The datasets used and/or analysed during the current study could be available from the corresponding author upon reasonable request if this is in line with current laws. No data can be shared that could identify the individual study participants.

Field-specific reporting

Please select the one below that is the best fit for your research. If you are not sure, read the appropriate sections before making your selection.

☒ Life sciences ☐ Behavioural & social sciences ☐ Ecological, evolutionary & environmental sciences

For a reference copy of the document with all sections, see nature.com/documents/nr-reporting-summary-flat.pdf

Life sciences study design

All studies must disclose on these points even when the disclosure is negative.

Sample size	This study is based upon a randomised controlled study including patients during 1986-1991 and included in the EBCTCG overview. No sample size calculation was performed for the present study. However, the original study had a calculated sample size of 500 patients, with a 90% power and an alpha-level of 5%, to detect an absolute difference of 15% regarding 5-year overall survival OS).
Data exclusions	Patients were excluded if no available PAM50/ROR score or IHC markers were available for the analyses we intended to perform. This is illustrated in the Flow chart.
Replication	N/A
Randomization	The randomisation was performed by the Regional Tumor Register in Lund and Linköping. Randomisation was performed in blocks of 8 according to the study protocol.
Blinding	No blinding.

Reporting for specific materials, systems and methods

We require information from authors about some types of materials, experimental systems and methods used in many studies. Here, indicate whether each material, system or method listed is relevant to your study. If you are not sure if a list item applies to your research, read the appropriate section before selecting a response.

Materials & experimental systems

n/a	Involved in the study	
☒	☐	Antibodies
☒	☐	Eukaryotic cell lines
☒	☐	Palaeontology and archaeology
☒	☐	Animals and other organisms
☐	☒	Human research participants
☐	☒	Clinical data
☒	☐	Dual use research of concern

Methods

n/a	Involved in the study	
☒	☐	ChIP-seq
☒	☐	Flow cytometry
☒	☐	MRI-based neuroimaging

Human research participants

Policy information about studies involving human research participants

Population characteristics	The patients in this study were included in the SBII:2pre study, which randomised 564 premenopausal women between 2 years of adjuvant tamoxifen or no systemic treatment.
Recruitment	Two coordinating centres including 20 hospitals participated in the study: the South Eastern (Oncological Centre Lund) and Southern (Oncological Centre Linköping) Health Care Regions. The coordinating centres crosschecked eiligibility according to a study specific report form which was collected for all randomised patients.
Ethics oversight	Approval was given by the ethical committees in Lund and Linköping, Sweden. The ethical committees approved oral informed consent by the patients verified by a written signature by the investigator in the report form which was sent to the coordinating center. At the time when the trial was approved and started, written consent was not considered ethically acceptable by the ethical boards in Sweden, but was thought to disturb the trustful patient-doctor relationship; thus, only oral consent was accepted and patients were orally informed.<br>The follow-up study was approved by the ethical committee of Lund (Dnr LU 2015/350) for extended follow-up as well as for genomic analysis (Dnr LU 2017/97). Biobank approval was cleared for all involved pathology departments.

Note that full information on the approval of the study protocol must also be provided in the manuscript.

Clinical data

Policy information about clinical studies

All manuscripts should comply with the ICMJE guidelines for publication of clinical research and a completed CONSORT checklist must be included with all submissions.

Clinical trial registration	This trial is registered in the ISRCTN database, trial ID: ISRCTN12474687.
Study protocol	Attached as a translated Supplementary Document
Data collection	Events (endpoint BCFi and RFi) were retrieved from central registries and medical charts (data cut-off Nov 30 2016). Events (endpoint OS) were retrieved from the Swedish Causes of Death Register (data cut-off 10 Dec, 2020).<br>Archival formalin-fixed paraffin-embedded (FFPE) tissues (n=520), including primary breast tumours from the study participants, were collected and RNA extracted for gene expression analyses.
Outcomes	The primary endpoints were BCFi including any of the following first events: local, regional, or distant recurrence; contralateral breast cancer (invasive or ductal cancer in situ); or breast cancer-related death according to the DATECAN recommendation. The secondary endpoint was OS. In sensitivity analysis, we additionally reported on RFi excluding contralateral breast cancer as events.
